# Visual Field Estimation in X-Linked Retinitis Pigmentosa Associated with Retinitis Pigmentosa GTPase Regulator (*RPGR*) from Image Analysis Using Artificial Intelligence

**DOI:** 10.1016/j.xops.2025.101033

**Published:** 2025-12-08

**Authors:** Malena Daich Varela, William Woof, Yathusha Kumarasamy, Matthias Monhart, Lynn Kandakji, Gunjan Naik, Pallavi Bagga, Alan Wilter Sousa, Dun Jack Fu, Catey Bunce, Konstantinos Balaskas, Nikolas Pontikos, Michel Michaelides

**Affiliations:** 1 Moorfields Eye Hospital, London, United Kingdom; 2 UCL Institute of Ophthalmology, University College London, London, United Kingdom; 3 Haag-Streit AG, Bern, Switzerland; 4 London School of Hygiene and Tropical Medicine, London, United Kingdom

**Keywords:** AI, Genetics, Retina, Retinitis pigmentosa, Visual field

## Abstract

**Purpose:**

To develop an efficient approach to estimating visual field (VF) in patients with X-linked retinitis pigmentosa (RP) based on macular OCT scans.

**Design:**

Retrospective analysis of patients who were enrolled in a natural history study at Moorfields Eye Hospital (London, United Kingdom).

**Subjects:**

Male patients with genetically confirmed retinitis pigmentosa GTPase regulator (*RPGR*)-associated RP.

**Methods:**

Visual field raw data were exported and analyzed including Visual Field Modeling and Analysis software. Retinal imaging consisted of OCT macular scans. Paired imaging and VF data acquired within a 1-month range were jointly analyzed. Artificial intelligence (AI) was used to automatically segment and quantify macular ellipsoid zone width (EZW), and ellipsoid zone area (EZA).

**Main Outcome Measures:**

Functional parameters from static VF testing such as mean sensitivity (MS) and Hill of Vision analysis that included total volume (V_TOT_), volume of central 20° (V_20_), and volume of central 30° (V_30_) were predicted from EZW and EZA.

**Results:**

Patient age ranged from 5 to 55 years old at baseline. A total of 332 OCT-VF pairs were analyzed. Ellipsoid zone area had the highest conditional R^2^ (R^2^c) and most significant associations with MS and V_20_. There were significant associations between MS and EZW (*P* = 0.00176), and MS with EZA (*P* = 0.0009).

**Conclusions:**

This study showed that AI enables efficient acquiring of large amounts of structural OCT parameters, facilitating research and structure-function predictions. The cohort included patients with a wide range of disease severity and statistical significance was achieved with parameters representing a wide range of VF, proving that this method can be applied for patients with milder disease.

**Financial Disclosure(s):**

Proprietary or commercial disclosure may be found in the Footnotes and Disclosures at the end of this article.

Inherited retinal disorders (IRDs) are a varied group of conditions with a wide range of effects on the visual experience.^1^ It is reported that >5 million people worldwide are affected with an IRD, or approximately 1 every 2000 individuals.^2^^,^^3^ They are the leading cause of blindness and visual disability in the working-age population of Australia and the United Kingdom.^4^^,^^5^ The most common type of IRD is retinitis pigmentosa (RP), a rod-cone dystrophy with a prevalence of roughly 1 in 3000 individuals.^6^

Patients affected by RP typically present with night blindness and peripheral field loss. The disease is often monitored by multimodal testing such as retinal imaging, visual field (VF) testing, visual acuity, and contrast sensitivity.^1^^,^^7^ By doing multiple tests, the aim is to capture and monitor both retinal structure and function as the disease slowly progresses. Although this approach is ideal to comprehensively capture patients’ visual experience, often not all tests are available owing to cost, time, and staff readiness, especially in the public health environment.

Visual field testing is a key parameter in visual assessment, necessary to define levels of visual impairment by public health organizations such as the World Health Organization.^8^ However, in practice, it can be time consuming, needing multiple attempts to reach a reliable result, more so in children and patients with severe visual impairment. Recent advances have been made to predict VF based on structural parameters such as OCT in patients with glaucoma.^9^^,^^10^ An initial study in 18 eyes with RP found a good correlation between kinetic perimetry and OCT ellipsoid zone width (EZW),^11^ and a more recent approach used ultra-widefield fundus autofluorescence images to estimate visual acuity, VF, and central retinal sensitivity in eyes with RP.^12^

X-linked RP (XLRP) accounts for up to 15% of all RP cases,^13^ usually having a rather severe presentation in males, being symptomatic in childhood and rapidly progressing to severe visual impairment by the fourth decade of life.^14^ Variants in retinitis pigmentosa GTPase regulator (*RPGR*) are the predominant cause of XLRP, accounting for 70% to 80% of cases.^15^ Retinitis pigmentosa GTPase regulator-associated RP follows a classical rod-cone dystrophy pattern, and structure-function correlations have been attempted in the past, where fundus autofluorescence findings were found highly correlated with electrophysiology testing.^16^^,^^17^ Tee et al^18^ also found statistically significant correlation between OCT and VF parameters on 53 eyes of 28 patients, with EZW being a stronger predictor than ellipsoid zone area (EZA).

In this study, we applied artificial intelligence (AI)-based segmentation algorithms to automatically quantify the transfoveal EZW and macular EZA in a large number of OCT scans of patients with *RPGR*-associated RP enrolled in a natural history study.^19^ These structural data were linked with various parameters from static VF testing done simultaneously or at near time point to the retinal imaging. By analyzing this comprehensive data, we present a valuable method to estimate the VF of patients with RP, based on easily acquired, fast OCT scans.

## Methods

Fifty-nine male patients with genetically confirmed *RPGR*-associated IRD participated in a natural history study that took place in Moorfields Eye Hospital (London, United Kingdom). Informed consent was obtained from all patients, ethical approval was provided by the local ethics committee, and the study honored the tenets of the Declaration of Helsinki.

**Figure 1:**
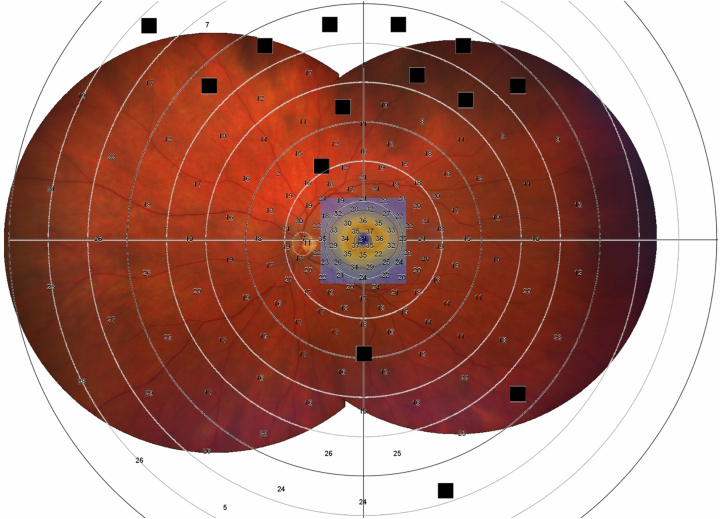
Overlap of widefield fundus image montage of patient with *RPGR*-related retinitis pigmentosa, and the area of the macular OCT scan that is indicated by the blue square (which is what is used to infer loss of the ellipsoid zone), and the custom visual field grid of 185 stimuli. Within the OCT area which corresponds to 30°, the red circle corresponds to 20°. *RGPR* = retinitis pigmentosa GTPase regulator.

The patients had consistent OCT (Heidelberg Spectralis, Heidelberg Engineering, Inc) and Octopus static perimeter VF (Haag-Streit AG) on every visit (every 6–12 months for 5 years) with both eyes separately (Fig 1).

### Retinal Imaging

Patients were dilated using 1% tropicamide and 2.5% phenylephrine hydrochloride. OCT imaging was acquired by site-certified technicians. Macular OCT volume scans were acquired with the fovea being in the center of the scan. OCT acquisition parameters were a 20° × 20° volume scan with 193 B-scans, ART 12, high-resolution horizontal imaging, and enhanced depth imaging capturing 25 sections was also captured. A reduced imaging protocol was used for younger patients.

### Image Analysis Using AI

**Figure 2:**
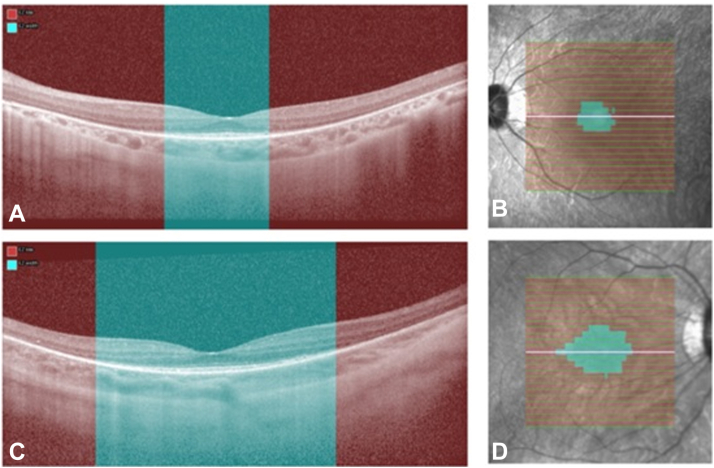
The automated segmentation of the ellipsoid zone loss (EZ loss) in red to produce the EZ width (EZW) (**A, C**) and the area (EZA) (**B, D**) in blue. Scans **A** and **B** represent a patient with a smaller area of remaining EZ, whereas scans **C** and **D** represent a patient with a larger area of remaining EZ.

An automated segmentation algorithm fined-tuned for IRDs (AIRDetect-OCT) was used to detect the foveal scan and measure transfoveal EZW and macular EZA.^20^^,^^21^ The model was externally validated for transfoveal EZW on 17 *RPGR* cases with a model-grader DICE of 0.75. For each horizontal OCT volume, an automatically segmented mask of the extent of ellipsoid zone (EZ) loss was automatically generated by the AI algorithm. Ellipsoid zone width was obtained for each B-scan by calculating the proportion of A-scan columns containing ≥1 pixel of EZ loss mask (Fig 2). This was then multiplied by the total width of the B-scan in millimeters. To calculate EZA, the EZW for each individual B-scan were summed and then multiplied by the B-scan spacing. Where multiple scans were available for a given appointment, the mean value over eligible scans was taken.

### VF Testing

Patients were tested undilated. A custom grid of 185 stimuli Goldmann size V was used, testing first the central 30° field of vision and then 180°, after a break (Fig 1). Fixation was monitored by the site-certified technician throughout the test as well as the reliability factor of the testing. Reliability factor value is the cumulation of the false-positive and false-negative catch trials used by Octopus software, EyeSuite. Visual field with reliability factor >30 were deemed unreliable and excluded from analysis. Parameters such as mean sensitivity (MS), mean deviation (MD), diffuse defect (DD), and local defect were considered for analysis.^22^ In brief, MS represents the patient’s average sensitivity to light, MD the average VF loss, DD the diffuse defect, and local defect the mean local defect. Visual field degrees estimation based on MS values was extracted from Haag-Streit AG Perimetry Simulation, where for a size V stimulus for an adult of 20 years old, a 20° field corresponded to ∼25.3 decibels (dB) MS, a 10° field to ∼9.1 dB MS, a 5° field to ∼3.3 dB MS, and a 1° field to ∼0.65 dB MS.

Raw data were exported from EyeSuite and uploaded into a Visual Field Modeling and Analysis software, to obtain 3-dimensional Hill of Vision parameters. Total Hill of Vision (V_TOT_), central 20° Hill of Vision (V_20_), and central 30° (V_30_) were analyzed. Given that OCT images acquired in this study cover the central vision, a detailed analysis processing raw VF parameters using a custom R script was also conducted to calculate the MS of the central 20° (MS_20_) and 30° of the VF (MS_30_).

### Outcome Measures and Statistical Analysis

The primary outcome of interest was MS and secondary outcomes included MS_20_, MS_30_, V_TOT_, V_20_, and V_30_ (Table S1, available at www.ophthalmologyscience.org). The association between structural metrics and the aforementioned was evaluated.

Single eye imaging (macular OCT) and VF assessments that took place within a 1-month range were linked together. To analyze this data, linear mixed-effects models was used to account for the repeated measurements from both eyes and multiple visits per patient. All analyses were performed using R version 4.4.1 (The R Foundation for Statistical Computing), and the lme4 package was used for mixed-effects modeling. To determine the best-fitting models and compare between models, Akaike information criterion (AIC) and *P* values were taken into account. The threshold for statistical significance was set at *P* < 0.05. All *P* values are exploratory or hypothesis-generating and correction for multiple comparisons was not applied.^23^

## Results

### Data Set

Initially, 467 pairs of OCT + VFs were identified. Fifteen VF were excluded owing to having a reliability factor >30, 38 corresponded to patients that had cone-rod dystrophy or sector RP phenotype, and 82 OCTs were not linked to VF testing owing to more than a month gap between assessments. The final data set included in this study were 332 pairs of retinal OCT and corresponding VFs of right and left eyes of 49 patients from 5 to 55 years old at baseline, with up to 14 pairs per patient (right and left eyes, during 7 visits taking place across 5 years).

### Baseline Characteristics

Each feature’s characteristics are described in Table 2. Fourteen VFs (4%) of young patients had a MS of >19 dB, corresponding to >20° VF. Maximum EZW was 5.85 mm, which is equivalent to a 15° to 20° macular scan, and within the 6 mm width of the scan dimensions.

**Table 2:** Functional and Structural Parameters in the Cohort

Type of Parameter	Parameter	Mean ± SD	Median
Visual fields (functional features)	MS (dB)	7.8 ± 5.6	5.8
	MD (dB)	22.8 ± 5.3	24.8
	DD (dB)	17.9 ± 6.1	18.2
	LD (dB)	8.2 ± 3.1	7.7
	MS_30_ (dB)	9.1 ± 6.6	7.2
	MS_20_ (dB)	10.5 ± 7.1	8.9
	V_TOT_ (dB-sr)	23.5 ± 19.5	18.2
	V_30_ (dB-sr)	6.4 ± 5.5	4.3
	V_20_ (dB-sr)	3.6 ± 2.7	2.9
Retinal scans (structural features)	EZ width (mm)	1.4 ± 1.5	0.8
	EZ area (mm^2^)	5.3 ± 6.6	2.7

dB = decibels; DD = diffuse defect; EZ = ellipsoid zone; LD = local defect; MD = mean deviation; MS = mean sensitivity; SD = standard deviation; V_20_ = volume of central 20°; V_30_ = volume of central 30°; V_TOT_ = total volume.

### Data Analysis

**Figure 3:**
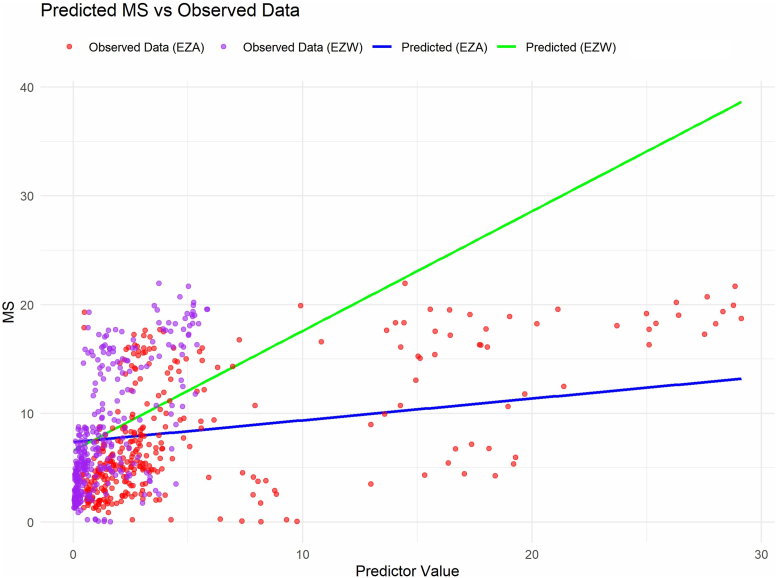
Visual field predictions based on ellipsoid zone width (EZW) and area (EZA). Ellipsoid zone width line-of-best-fit is diagrammed in green line and for EZA in blue. MS = mean sensitivity.

The linear mixed-effects model for MS revealed a significant effect of EZW on MS, with an estimated coefficient of 1.1 (standard error [SE] = 0.32, *P* = 0.00176). For EZA, the coefficient was 0.2 (SE = 0.06, *P* = 0.0009). Although with substantial patient-level variance, the line-of-best-fit in Figure 3 may point at a foveal EZW of 1 mm corresponding to around 7.7 dB MS, and a monocular field between 5° and 10°, and a 2 mm EZW to around 10° with that eye.

Conditional R^2^ (R^2^c)—variable that captures the proportion of variance in MS explained by both fixed (EZW or EZA) and random effects (repeated visits and measurements from both eyes per patient)—was 92.7% for the EZW model and 92.2% for the EZA model. Model performance was also compared using the AIC, which was 1470.34 for the EZW model and 1464.92 for the EZA model. Although the difference is modest, the lower AIC suggests that EZA may serve as a slightly better predictor of MS.

Random effects analyses showed significant variability at both patient and eye levels. The random intercept for patients had a variance of 24.9551 (standard deviation = 4.9955), indicating substantial variability in MS across patients, even after accounting for fixed effects. This suggests that there are individual differences that contribute meaningfully to the observed variability in MS, even after adjusting for EZA or EZW. Additionally, the random intercept for right/left eye had a smaller variance of 0.0354 (standard deviation = 0.1881), indicating a modest correlation between repeated measurements within the same eye.

When testing associations with MS_30_, EZW and EZA showed significant associations, with a coefficient of 0.67 (SE = 0.2564, *P* = 0.009) for EZW and 0.26 (SE = 0.0698, *P* = 0.00027) for EZA. Ellipsoid zone area provided a better fit to the data (AIC 1572.66 vs. 1576.15), with both models having similar R^2^c (∼0.92).

For MS_20_, both EZW and EZA showed significant associations. For EZW, the estimate was 1.3064 (*P* = 3.77e-06), whereas for EZA, the estimate was 0.3710 (*P* = 1.64e-06). The model with EZA exhibited slightly better fit indices (R^2^c = 0.9069) compared with the model with EZW (R^2^c = 0.8923), although both models showed strong predictive power. The AIC values for both models were similar (EZW 1638.93, EZA 1638.41). Further associations between MD, DD, local defect, V_TOT_, V_30_, and V_20_ are detailed in Table 3.

**Table 3:** Linear Regression Analysis of Functional and Structural Parameters

Parameter	EZ Width (mm)				EZ Area (mm^2^)			
	*P* Value	R^2^c	R^2^m	AIC	*P* Value	R^2^c	R^2^m	AIC
**MS (dB)**	**0.00176**	92.7%	0.63%	1470.34	**0.0009**	92.2%	5.96%	1464.91
**MD (dB)**	0.274	91.7%	0.47%	1462.95	**0.0007**	90.9%	6.86%	1456.922
**DD (dB)**	**0.00989**	88.3%	3.4%	1600.01	**2.05e-07**	89.1%	18.17%	1583.74
**LD (dB)**	**0.0861**	78.9%	2.14%	1381.56	**0.00253**	81.1%	8.05%	1378.183
MS_30_**(dB)**	**0.009**	91.6%	2.72%	1576.15	**0.00027**	91.9%	7.32%	1572.658
MS_20_**(dB)**	**3.77e-06**	89.2%	7.59%	1638.92	**1.64e-06**	90.7%	16.1%	1638.407
V_TOT_**(dB-sr)**	0.862	88.4%	0.01%	2406.20	**0.0392**	87.2%	3.26%	2405.198
V_30_**(dB-sr)**	0.775	92.2%	8.72%	1456.24	**0.0451**	91.6%	13.79%	1455.355
V_20_**(dB-sr)**	**0.0003**	89.4%	6.27%	987.09	**2.80e-05**	90.5%	10.81%	984.87

AIC = Akaike information criterion; dB = decibels; DD = diffuse defect; EZ = ellipsoid zone; LD = local defect; MD = mean deviation; MS = mean sensitivity; R^2^c = conditional R^2^; R^2^m = marginal R^2^; V_20_ = volume of central 20°; V_30_ = volume of central 30°; V_TOT_ = total volume.

Statistically significant *P* values are in bold.

## Discussion

### Background

Visual field constriction is one of the most disabling symptoms of RP, often causing patients to trip, bump into things, miss objects that fall within the scotomata’s region, and lose independence, being highly associated with perceived visual disability.^24^ Furthermore, patients with RP often struggle to convey their visual experiences to friends and family, who may not fully understand their challenges.^25^ Hence, a frequent question posed by patients and their loved ones is how much VF the patient maintains, to have a practical understanding of the severity of the condition. Visual field testing is often not readily available for all patients; however, the answer is deferred until appropriate testing can be done. With the analysis herein, we aimed at facilitating the answer for those patients, providing ophthalmologists with an easy tool that can estimate the VF of a patient with RP based on macular EZW and EZA.

OCT and EZ parameters have been known to correlate with VF in patients with RP cross-sectionally and longitudinally for over a decade.^18^^,^^26^^,^^27^ Tee et al^18^ found a stronger correlation between EZW and VF parameters, compared with EZA, in 38 subjects with *RPGR*-associated RP. Previous groups such as Hara et al^28^ found a statistically significant association between EZW and macular sensitivity of the central 10° of the retina, assessed by Humphrey static perimeter. In their cohort, an EZW of 2000 μm corresponded with a macular sensitivity between 15 and 20 dB. Battu et al^29^ found a significant correlation between total and outer retinal thickness and retinal sensitivity on microperimetry. Yoon and Yu^30^ found a significant association between EZA and EZW and kinetic VF area; however, no clear practical correlation was provided. Asahina et al^31^ tested the association between EZA, Humphrey VF and MP-3 microperimetry, finding a significant association between EZA and MP-3 only, with an EZA of 2 mm^2^ corresponding to around 9 dB in MP-3. Funatsu et al^32^ found a strong correlation between outer retinal thickness and retinal sensitivity with MP-3, which got weaker as it went away from the fovea. Lastly, Smith et al^33^ analyzed the association between OCT and VF parameters in patients with autosomal dominant RP and did not find significant associations between EZW and MS or V_TOT_.

### Results Applicability

The focus of this project was to create an easy tool to predict VF in clinical practice. Ellipsoid zone area showed the best and most significant associations with MS, MD, DD, MS_30_, MS_20_, and V_20_, with low *P* values and high R^2^c (Table 3). The incorporation of automatic EZA measurement into OCT devices could be useful to triage patients, identifying those who may be reaching the threshold of driving, or to provide insights for patients who do not want or cannot do a VF. A faster/shorter VF test could also be attempted in those with a predicted small VF remaining, making it more accessible for those who need it for decision-making purposes. Alternatively, by manually measuring the EZW at the foveal scan and using the equation presented, we are able to provide the patient with an estimation of how much VF is remaining in degrees or in percentage format (Fig 2).

Our results are similar to those of Fischer et al,^11^ and interestingly in our cohort, an EZW of 2000 μm corresponded with less MS than in Hara et al^28^ (nearly 10 dB vs 15–20 dB), who analyzed a majority of patients with simplex RP. An EZA of 2 mm^2^ yielded similar results in our cohort (∼7.8 dB) compared with Asahina et al^31^ (∼9 dB), who included mostly not-genetically confirmed patients. It is probable that the different VF devices used across studies contribute to these discrepancies, and also the variable RP subtypes; for example, patients with XLRP have a faster progression than other types of RP,^34^ with functional impairment preceding structural deficit.^35^ Future studies evaluating if an EZW of 1 mm corresponds to more or less VF depending on the genotype would be useful to further clarify this hypothesis.

The use of AI-based segmentation tools enables large-scale data analysis where human annotation would otherwise be too laborious and time consuming as to make it impractical, especially in the case of OCT volumes where each B-scan must be annotated individually, possibly by ≥2 graders. Particularly in the field of IRD, where the phenotype is characteristically highly variable, the inclusion of more patients from as broad a range of ages and disease severity is essential.^36^^,^^37^ With the inclusion of AI, we were able to analyze a large volume of structural data and draw correlations with meaningful impact on clinical practice. In the future, Spectralis may directly provide reliable automated measurements of EZA and EZW along with the VF estimation proposed herein, this being another example in which AI and digital health combined with knowledgeable oversight can be used for improving healthcare.

**Figure 4:**
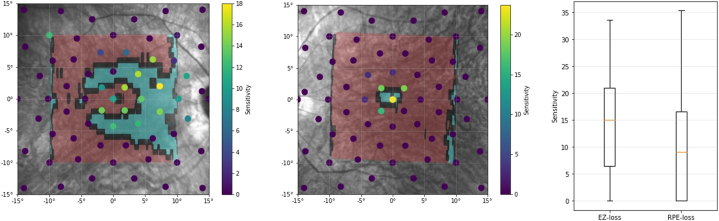
Visual field examination overlaid on example infrared enface images, with regions of ellipsoid zone (EZ) loss (in green) and retinal pigment epithelium (RPE) loss (in red) projected from the corresponding OCT imaging. Patients seem to retain some vision even in regions of the retina with possible EZ loss or irregular/patchy EZ layer.

Patients with RP often have an initial ring-shaped scotoma in the midperiphery, preserving the far end of their VF, and only retain a central island of VF over time (Fig 4).^1^^,^^38^ By analyzing widefield Octopus VF data, we aimed at capturing a comprehensive VF experience, including the remnant peripheral field in younger/mildly affected patients. However, these peripheral values impact the overall MS of the test, hence the MS to central VF degrees estimation is only an approximation.

### Strengths and Limitations

Our study’s strengths are the large number of genetically confirmed individuals with a single genetic basis followed for a long period of time, and the standardized testing acquired by the same machines on the same settings over time. Some of the study limitations include the age and severity variability in our sample, reflecting the disease natural history.^39^ Also, the AI algorithm could be further improved to capture other parameters such as retinal thinning and increase the accuracy of EZA and EZW detection. There were also differences in follow-up between patients and device operators. Factors such as unknown distance between patient and device and axial length may also affect the OCT measurements; the values herein should be considered as an estimation or a broad reference point. Our study was also limited to patients with XLRP, whereas other *RPGR* phenotypes are likely to exhibit different correspondence between EZA/EZW and VF. Future work testing the correlation proposed herein in patients with other types of RP and IRD is needed to validate this hypothesis in other genotypes.

### Conclusions

This study provides an evidence-based method to estimate the VF of patients with XLRP, where an EZW of 1000 μm corresponds to a monocular field between 5° and 10°, and a 2000 μm EZ width to around 10° with that eye. Additional large-scale studies are necessary to confirm if the same EZW corresponds to different degrees of VF loss depending on the genotype. The findings of this study offer a valuable tool for patients and healthcare providers.
