# Supplementary material for: Visual Field Estimation in X-Linked Retinitis Pigmentosa Associated with Retinitis Pigmentosa GTPase Regulator (RPGR) from Image Analysis Using Artificial Intelligence
Source: Ophthalmol Sci. 2025 Dec 8;6(3):101033. doi: 10.1016/j.xops.2025.101033 (PMC12914202; doi:10.1016/j.xops.2025.101033)
Supplement: Supplementary Table S1 [file mmc1.pdf]

Table S1: Definition of functional parameters

**Function parameter**	**Definition**	**Units**
RF	Reliability Factor	NA
MS	Mean Sensitivity	dB
MD	Mean Deviation	dB
DD	Diffuse Defect	dB
LD	Local Defect	dB
HOV	Hill of Vision	NA
V_{TOT}	Total Hill of Vision	dB-sr
V_{20}	Central 20° Hill of Vision	dB-sr
V_{30}	Central 30° Hill of Vision	dB-sr
VFMA	Visual Field Modelling and Analysis	NA
